# A Notch/STAT3-driven Blimp-1/c-Maf-dependent molecular switch induces IL-10 expression in human CD4^+^ T cells and is defective in Crohn´s disease patients

**DOI:** 10.1038/s41385-022-00487-x

**Published:** 2022-02-15

**Authors:** Jonas Ahlers, Andrej Mantei, Laura Lozza, Manuela Stäber, Frederik Heinrich, Petra Bacher, Thordis Hohnstein, Lutz Menzel, Simge G. Yüz, Daniel Alvarez-Simon, Anne Rieke Bickenbach, Carl Weidinger, Nadine Mockel-Tenbrinck, Anja A. Kühl, Britta Siegmund, Jochen Maul, Christian Neumann, Alexander Scheffold

**Affiliations:** 1 Department of Rheumatology and Clinical Immunology, Charité—Universitätsmedizin Berlin, Berlin, Germany; 2 Labor Berlin, Charité Vivantes GmbH, Berlin, Germany; 3 Cell Biology, Precision for Medicine GmbH, Berlin, Germany; 4 Central Lab Service, Max-Plack-Institute for Infection Biology, Berlin, Germany; 5 German Rheumatism Research Center (DRFZ) Berlin, Leibniz Association, Berlin, Germany; 6 Institute of Immunology, Christian-Albrechts-University of Kiel & UKSH Schleswig-Holstein, Kiel, Schleswig-Holstein, Germany; 7 Institute of Clinical Molecular Biology, Christian-Albrechts-University of Kiel, Kiel, Schleswig-Holstein, Germany; 8 Department of Microbiology, Infectious Diseases and Immunology, Charité – Universitätsmedizin Berlin, Berlin, Germany; 9 Translational Tumor Immunology, Max-Delbrück-Center for Molecular Medicine, Berlin, Germany; 10 Department of Gastroenterology, Infectious Diseases and Rheumatology, Campus Benjamin Franklin, Charité – Universitätsmedizin Berlin, Berlin, Germany; 11 Miltenyi Biotec B.V. & Co.KG, Bergisch-Gladbach, Nordrhein-Westfalen Germany; 12 iPATH, Campus Benjamin Franklin, Charité—Universitätsmedizin Berlin, Berlin, Germany; 13 Gastroenterologie am Bayerischen Platz, Berlin, Germany; 14 Present Address: Sanofi Pasteur, Sanofi-Aventis Deutschland GmbH, Berlin, Germany

## Abstract

Immunosuppressive Interleukin (IL)−10 production by pro-inflammatory CD4^+^ T cells is a central self-regulatory function to limit aberrant inflammation. Still, the molecular mediators controlling IL-10 expression in human CD4^+^ T cells are largely undefined. Here, we identify a Notch/STAT3 signaling-module as a universal molecular switch to induce IL-10 expression across human naïve and major effector CD4^+^ T cell subsets. IL-10 induction was transient, jointly controlled by the transcription factors Blimp-1/c-Maf and accompanied by upregulation of several co-inhibitory receptors, including LAG-3, CD49b, PD-1, TIM-3 and TIGIT. Consistent with a protective role of IL-10 in inflammatory bowel diseases (IBD), effector CD4^+^ T cells from Crohn’s disease patients were defective in Notch/STAT3-induced IL-10 production and skewed towards an inflammatory Th1/17 cell phenotype. Collectively, our data identify a Notch/STAT3—Blimp-1/c-Maf axis as a common anti-inflammatory pathway in human CD4^+^ T cells, which is defective in IBD and thus may represent an attractive therapeutic target.

## Introduction

The anti-inflammatory cytokine Interleukin (IL-) 10 plays a key role in the establishment of immune tolerance and prevention of immunopathology by limiting immune responses against harmless antigens and constraining excessive immunity against pathogens.^1,2^ In particular, IL-10 is essential for the regulation of intestinal immune homeostasis by assuring balanced immune control of the microbiota.^3^ In mice and humans, deficiencies in IL-10 or the IL-10 receptor result in early and severe intestinal inflammation.^3,4^ Similarly, polymorphisms related to the function of IL-10 or its receptor are associated with the development of inflammatory bowel disease (IBD) in humans.^3^

Importantly, virtually all cells of the innate and adaptive immune system can secrete IL-10.^1,5^ However, various murine T cell-specific knock-out models have established non-redundant functions for CD4^+^ T cell-derived IL-10 in maintaining immune homeostasis to intestinal commensal microbes and during the resolution phase of pathogen infections.^2^ Indeed, CD4^+^ T cell-specific loss of IL-10 in mice is sufficient to cause spontaneous development of intestinal inflammation.^6,7^

Initially, CD4^+^ T cell-derived IL-10 production has been attributed to specialized “regulatory” CD4^+^ T cell subsets, such as Foxp3^+^ regulatory T cells and T regulatory type 1 (Tr1) cells,^7–11^ which produce preferentially IL-10 but also effector cytokines at variable levels. However, it is now well established that also inflammatory effector CD4^+^ T cells can acquire IL-10 co-expression at least transiently,^12,13^ as a crucial intrinsic negative feedback mechanism for the self-limitation of inflammatory immune responses.^14–16^

Consistent with this ubiquitous expression, the signals that drive the differentiation of the various effector CD4^+^ T helper (Th) cell subsets also contribute to IL-10 expression, including IL-12 (STAT4) for Th1 cells, IL-4 (STAT6) for Th2 cells as well as IL-6, IL-21 (STAT3) and TGF-ß for Th17 cells.^17–21^ In addition, IL-27 (STAT1/3) has been shown to promote IL-10 expression by murine CD4^+^ T cells.^22–27^ In humans, type I interferon (IFN) (STAT1/3), which generally supports Th1 immunity, appears to co-stimulate CD4^+^ T cell-derived IL-10 production.^28–31^ In summary, all these IL-10 inducing signals predominantly drive inflammatory effector CD4^+^ Th cell programming. This suggests an additional layer of IL-10 regulation, which allows to precisely adjust its expression to the microenvironmental conditions and to switch from pro- to anti-inflammatory function.

Indeed, in murine CD4^+^ T cells we have identified the Notch signaling pathway to selectively elicit IL-10 expression.^32–35^ Notch-mediated IL-10 induction was dependent on the integration of inflammatory cytokine signals, including STAT4-dependent IL-12/IL-27 in Th1 cells.^32,35^ On the transcriptional level Notch and cytokine signaling converge on the transcription factors Blimp-1 and c-Maf, which have been described as master regulators of IL-10 in murine CD4^+^ T cells.^27,35–43^ However, in the human system, the role of Notch and cytokine co-signaling for IL-10 regulation during homeostasis or inflammation, as well as the identity of the underlying transcriptional networks, have remained poorly defined.^44,45^

Here, we demonstrate that activation of the Notch signaling pathway together with the STAT3-activating cytokines IFN-α, IL-6 and IL-21 selectively induced transient, c-Maf- and Blimp1-dependent expression of IL-10 in human naïve and all major memory/effector CD4^+^ T cell subsets. The Notch/STAT3-mediated acquisition of IL-10 expression was accompanied by the potent upregulation of several co-inhibitory receptors, such as CD49b, LAG-3, PD-1, TIM3 and TIGIT, indicating a global reprogramming towards a regulatory phenotype. Interestingly, we found that this specific IL-10-inducing pathway was impaired in Crohn’s disease patients. Thus, our data identify Notch/STAT3 as a universal molecular switch regulating anti-inflammatory functions in human CD4^+^ T cells as a potential target in chronic inflammation.

## Results

### Notch signaling integrates STAT3-dependent inflammatory cytokine cues to selectively induce IL-10 expression in human naïve and memory CD4^+^ T cells

The acquisition of immunosuppressive IL-10 production by effector memory CD4^+^ T cells represents an important self-regulatory function.^14,15^ However, in humans the extra- and intracellular pathways controlling IL-10 expression in effector memory CD4^+^ T cells are poorly defined.

**Fig. 1:**
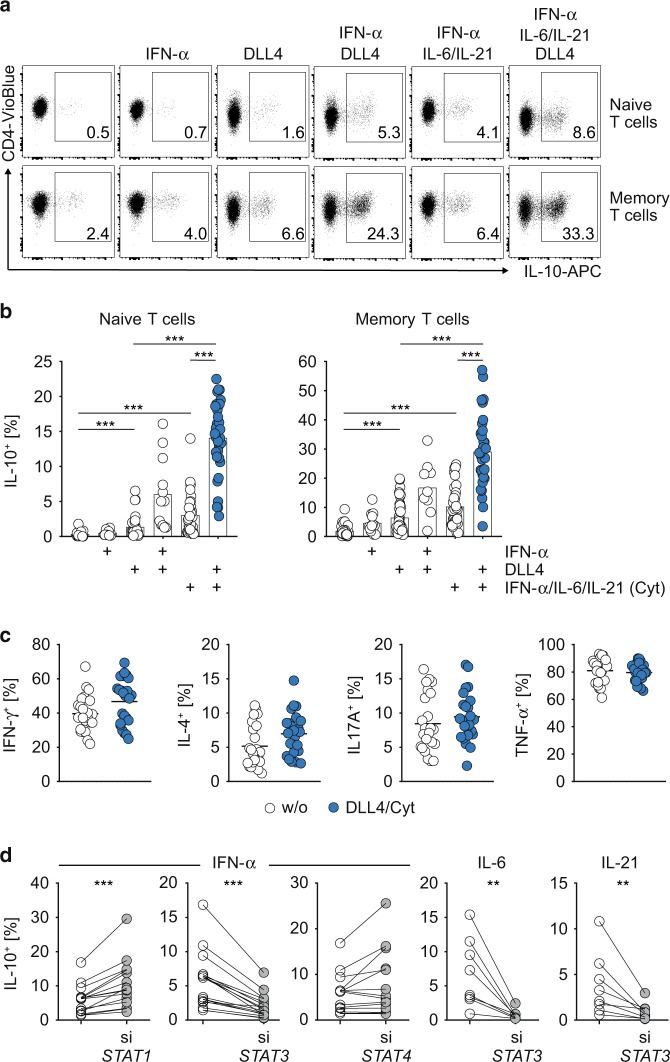
Selective induction of IL-10 in human naïve and memory CD4^+^ T cells by Notch/STAT3 signaling. **a** Expression of IL-10 by in vitro cultured human naïve (upper row) and memory (lower row) CD4^+^ T cells as measured by flow cytometry (pre-gated on viable lymphocytes, representative flow cytometric profiles). CD4^+^ T cells were cultured under indicated conditions for 120 h and afterwards restimulated with PMA/Ionomycin for optimal cytokine detection. **b** Quantification of IL-10^+^ cells among naive (left) and memory (right) CD4^+^ T cells. Each dot represents one healthy donor. If not indicated otherwise, significance refers to T cells cultivated without cytokines/DLL4. **c** Quantification of IFN-γ^+^, IL-4^+^, IL-17^+^ and TNF-α^+^ cells among memory CD4^+^ T cells after TCR-only (w/o) or DLL4/Cyt (Cyt = IFN-α/IL-6/IL-21) co-stimulation. **d** Naïve CD4^+^ T cells were transfected ex vivo with siRNA against indicated *STAT*s (gray circles) or with scrambled control siRNA (white circles) and subsequently activated and cultivated in vitro for 120 h with DLL4 and indicated cytokines. Each dot represents one healthy donor (*n* = 8–36). Two-tailed Wilcoxon, Mann–Whitney and unpaired *t* tests were performed (*P < 0.05; **P < 0.01; ***P < 0.001) to assess significance. Data are cumulative from up to twelve independent experiments.

Previously, the inflammatory cytokine IFN-α was reported to stimulate IL-10 production from human naïve CD4^+^ T cells and to promote the differentiation of IL-10-producing Tr1 cells.^28–31^ However, the immunomodulatory effect of IFN-α on human effector memory CD4^+^ T cells has remained largely unknown. Therefore, we directly tested the IL-10-inducing capacity of IFN-α on PBMC-derived effector memory (CD45RA^−^ R0^+^ CD31^−^) CD4^+^ T cells. As a control, we used naïve (CD45RA^+^ R0^−^ CD31^+^) CD4^+^ T cells in our analysis. Indeed, in line with previous reports, in vitro T cell receptor (TCR) stimulation of naïve CD4^+^ T cells with anti-CD3/CD28 in the presence of IFN-α resulted in a slightly increased frequency of IL-10-producing T cells as compared to TCR stimulation without IFN-α (Fig. 1a, b). Importantly, IFN-α stimulation also increased the frequency of IL-10^+^ cells among effector memory CD4^+^ T cells (Fig. 1a, b), corroborating its immunomodulatory role in human CD4^+^ T cells.

In previous studies, we identified the Notch signaling pathway to synergize with STAT4-activating cytokines in the induction of IL-10 expression in murine effector CD4^+^ T cells.^32,33^ Therefore, we asked whether activation of Notch in human CD4^+^ T cells also potentiates IL-10 expression. To this end, we co-stimulated naïve and memory CD4^+^ T cells in vitro with the Notch ligand Delta-like 4 (DLL4). Indeed, DLL4 co-stimulation significantly increased IL-10 production by both naïve and memory CD4^+^ T cells at levels comparable to IFN-α stimulation (Fig. 1a, b). Importantly, when combined with IFN-α, DLL4 co-stimulation resulted in a synergistic upregulation of IL-10 expression by naïve and memory CD4^+^ T cells (Fig. 1a, b).

In search of additional signals that promote IL-10 production by human CD4^+^ T cells, we treated IFN-α and IFN-α + DLL4 co-stimulated naïve and memory CD4^+^ T cells also with IL-6 and IL-21, since both cytokines were shown to support the differentiation of murine IL-10-producing T cells.^20,23,25,46^ Indeed, addition of IL-6 and IL-21 further increased the IFN-α-mediated IL-10 induction (Fig. 1a, b). More strikingly, the combination of IFN-α/IL-6/IL-21 [“cytokines (cyt)”] with DLL4 resulted in an approximately tenfold increase in the frequency of IL-10^+^ T cells as compared to TCR-only stimulated cells both in the naïve and memory compartment (Fig. 1a, b). Notably, other candidate cytokines tested, including IL-12, IL-23, IL-27 and TGF-β, did not show comparable synergy with DLL4 to augment IL-10 induction (data not shown). DLL4 + cytokine co-stimulation did not change the expression of inflammatory effector cytokines by memory CD4^+^ T cells, such IFN-γ, IL-4, IL-17 and TNF-α (Fig. 1c). From a mechanistical point of view, IL-10 induction by cytokine stimulation was solely dependent on STAT3, but not on STAT1 or STAT4, as evidenced by siRNA-mediated knock-down experiments (Figs. 1d, and S1).

Collectively, these data demonstrated that Notch signaling synergized with the inflammatory cytokines IFN-α, IL-6 and IL-21 to selectively induce IL-10 expression in human naïve and memory CD4^+^ T cells in a STAT3-dependent manner.

### IL-10 expression induced by Notch/STAT3 co-stimulation is transient and independent of CD4^+^ T helper cell polarization

**Fig. 2:**
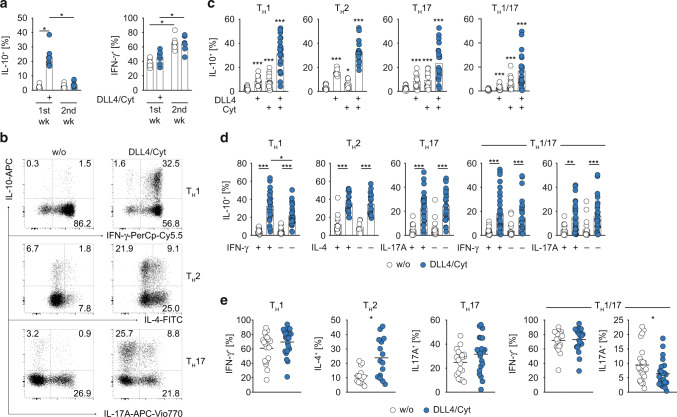
Notch/STAT3-induced IL-10 expression is transient and independent of CD4^+^ T_H_ cell specification. **a** Memory CD4^+^ T cells were cultivated w/ or w/o DLL4/Cyt (Cyt = IFN-α/IL-6/IL-21) co-stimulation for 120 h (1^st^ wk). Afterwards cells were rested and re-stimulated for additional 120 h (2^nd^ wk) without co-stimulation. After each round of stimulation, cells were restimulated with PMA/Ionomycin and subsequently stained for IL-10 and IFN-γ. Quantification of two independent experiments is shown. **b** FACS-isolated Th1, Th2 and Th17 cells were cultivated in vitro w/ or w/o DLL4/Cyt (Cyt = IFN-α/IL-6/IL-21) co-stimulation for 120 h and afterwards restimulated with PMA/Ionomycin for optimal cytokine detection. Representative flow cytometric profiles of IL-10 vs. IFN-γ, IL-4 and IL-17A expression are shown. **c** Quantification of IL-10^+^ cells among Th1, Th2, Th17 and Th1/17 cells cultivated in vitro w/ or w/o DLL4, Cyt or DLL4/Cyt (Cyt = IFN-α/IL-6/IL-21) co-stimulation. **d** Quantification of IL-10^+^ cells among IFN-γ^+/−^ (Th1, Th1/17), IL-4^+/−^ (Th2), IL-17A^+/−^ (Th17, Th1/17) cells cultivated in vitro w/ or w/o DLL4/Cyt (Cyt = IFN-α/IL-6/IL-21) co-stimulation. **e** Quantification of IFN-γ^+^, IL-4^+^ and IL-17A^+^ cells among FACS-isolated Th1, Th2, Th17 and Th1/17 cells, respectively, after in vitro cultivation and w/ or w/o DLL4/Cyt (Cyt = IFN-α/IL-6/IL-21) co-stimulation. Each dot represents one healthy donor (*n* = 7–32). Two-tailed Wilcoxon, Mann–Whitney and unpaired *t* tests were performed (*P < 0.05; **P < 0.01; ***P < 0.001) to assess significance. Data are cumulative from up to eight independent experiments.

It is still unclear whether acquisition of self-regulatory IL-10 expression by effector memory CD4^+^ T cells represents a durable change in the cytokine profile or rather a dynamic property within the functional plasticity of CD4^+^ T cells. To address this, we tested whether the heightened capability to express IL-10 by memory CD4^+^ T cells that were co-stimulated with DLL4 + STAT3 cytokines remained stable over time. As shown in Fig. 2a memory CD4^+^ T cells stimulated in the presence of DLL4 + STAT3 cytokines lost the capacity to express IL-10 after one additional week of culture in the absence of DLL4 + STAT3 cytokines, while IFN-γ expression was stably maintained. Thus, the capacity to express IL-10 upon a single round of Notch/STAT3 co-stimulation was transient and remained conditional on co-stimulatory signals.

Next, we wondered whether the ability to acquire IL-10 expression was confined to a distinct subset of memory CD4^+^ T cells and/or linked to a specific T helper (Th) cell differentiation program. Therefore, we FACS-purified the major Th cell subsets Th1, Th2 and Th17 cells directly from PBMCs based on their subset-specific pattern of chemokine receptor expression (Th1: CXCR3^+^, Th2: CCR4^+^, Th17: CCR6^+^CCR4^+^) (Fig. S2a).^47^ All populations exhibited a subset-specific signature cytokine profile after short-term ex vivo stimulation with PMA/Ionomycin, confirming their Th1, Th2 and Th17 cell identity (Fig. S2b).

When stimulated in vitro in the presence of DLL4 or STAT3 cytokines or both, all Th cell subsets upregulated IL-10 expression (Fig. 2b, c). Again, DLL4 + STAT3 cytokine co-stimulation elicited the strongest IL-10 response as compared to DLL4 or cytokine stimulation alone (Fig. 2b, c). Importantly, in all Th cell subsets, IL-10 expression was equally induced in effector cytokine positive and negative cells (e.g. IFN-γ^+^ vs. IFN-γ^−^ Th1 cells) (Fig. 2b, d), demonstrating that IL-10 induction by DLL4 + STAT3 cytokine co-stimulation was largely uncoupled from the expression of CD4^+^ Th cell subset-defining cytokines. Consistent with this, DLL4 + STAT3 cytokine co-stimulation only marginally altered the frequency of IFN-γ producers among Th1 cells and IL-17A producers among Th17 cells (Fig. 2e). Only Th2 cells responded with significantly elevated frequencies of IL-4-producing cells upon DLL4 + STAT3 cytokine co-stimulation (Fig. 2e).^48^ Importantly, our co-stimulation protocol also instructed IL-10 production by polyfunctional IFN-γ/IL-17 co-expressing CD4^+^ Th cells (termed Th1/17 cells) (Figs. 2c, d, e), which have been described as a potential pathogenic subset in chronic inflammatory diseases.^49^

Overall, these data showed that Notch and STAT3-activating cytokines synergized to act as an universal molecular switch to transiently induce IL-10 production in human effector memory CD4^+^ T cells irrespective of their polarization status.

### Notch/STAT3 co-stimulation induces a Tr1 cell phenotype in human memory CD4^+^ T cells

**Fig. 3:**
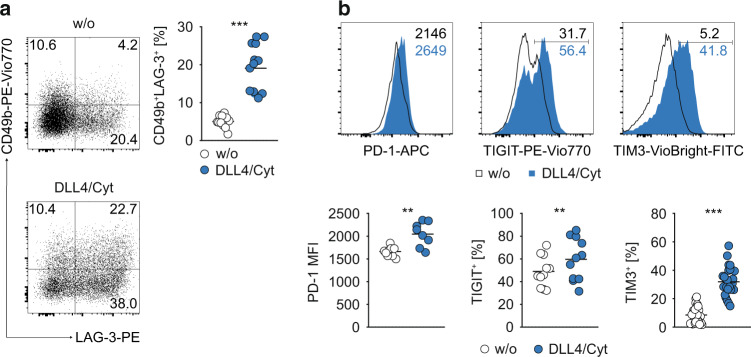
Notch/STAT3 co-stimulation enhances expression of several co-inhibitory receptors on human effector memory CD4^+^ T cells. **a** FACS-purified human effector memory CD4^+^ T cells were cultivated in vitro w/ or w/o DLL4/Cyt (Cyt = IFN-α/IL-6/IL-21) co-stimulation. Representative flow cytometric profiles of CD49b vs. LAG-3 expression (left) and quantification of CD49b^+^LAG-3^+^ co-expressing cells (right) are shown. **b** Expression of PD-1, TIGIT and TIM3 by effector memory CD4^+^ T cells after cultivation and co-stimulaton w/ or w/o DLL4/Cyt (Cyt = IFN-α/IL-6/IL-21). Representative histograms (upper row). Quantification of PD-1 MFI, TIGIT^+^ and TIM3^+^ cells among effector memory CD4^+^ T cells (lower row) is shown. Each dot represents one healthy donor (*n* = 8–26). Two-tailed paired *t* test was performed (**P < 0.01; ***P < 0.001) to assess significance. Data are cumulative from up to seven experiments.

It is not well understood whether acquisition of self-regulatory IL-10 expression by inflammatory effector memory CD4^+^ T cells merely reflects a change in cytokine expression or if memory CD4^+^ T cells can undergo a more global reprogramming towards an anti-inflammatory phenotype. To address this question, we measured the expression of several co-inhibitory surface receptors associated with an immunosuppressive phenotype.^50^ Indeed, DLL4 + STAT3 cytokine co-stimulation of memory CD4^+^ T cells resulted in a striking upregulation of CD49b and LAG-3 (Fig. 3a), two markers, which were previously shown to specifically identify murine and human IL-10-producing Tr1 cells.^51^ In addition, we also detected a significantly increased expression of negative costimulatory molecules (“checkpoints”) PD-1, TIM-3 and TIGIT upon DLL4 + STAT3 cytokine co-stimulation as compared to control stimulated cells (Fig. 3b). To confirm the suppressive function, we tested supernatants derived from memory CD4^+^ T cells stimulated with CD3/28 plus or minus DLL4/STAT3 cytokines for their effect on HLA-DR and CD40 induction in LPS-stimulated monocytes. Control supernatants strongly upregulated HLA-DR and CD40 (Fig. S3). In contrast, supernatants from DLL4/STAT3-cytokine co-stimulated cells did not increase HLA or even suppressed CD40, which was both dependent on IL-10, as shown by anti-IL-10R antibody blockade (Fig. S3)

Thus, in addition to IL-10, human memory CD4^+^ T cells upregulated the expression of several co-inhibitory receptors upon co-stimulation with Notch and STAT3 cytokines and acquired a suppressive Tr1 cell phenotype based on LAG-3 and CD49b co-expression.

### Transcription factors Blimp-1 and c-Maf synergistically control IL-10 expression downstream of Notch/STAT3 co-stimulation

**Fig. 4:**
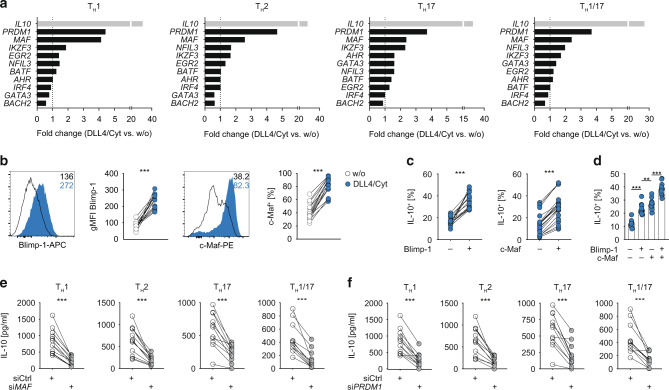
Blimp-1 and c-Maf are induced and jointly control IL-10 expression downstream of Notch/STAT3 signaling. **a** High-throughput microfluidic real-time PCR for indicated genes of FACS-purified Th1, Th2, Th17 and Th1/17 co-stimulated w/ or w/o DLL4/Cyt (Cyt = IFN-α/IL-6/IL-21) for 24 h. Fold change (DLL4/Cyt vs. w/o) is shown. Data are mean values from six different donors from two different experiments. **b** Expression of Blimp-1 and c-Maf by FACS-isolated effector memory CD4^+^ T cells after cultivation and co-stimulation w/ or w/o DLL4/Cyt (Cyt = IFN-α/IL-6/IL-21) for 48 h. Representative histograms (left) and quantification of gMFI of Blimp-1 or c-Maf^+^ cells (right). **c** Quantification of IL-10^+^ cells among Blimp-1^+/−^ or c-Maf^+/−^ cells. **d** Quantification of IL-10^+^ cells among Blimp-1^−^c-Maf^−^, Blimp-1^+^c-Maf^−^, Blimp-1^−^c-Maf^+^ and Blimp-1^+^c-Maf^+^ cells. **e**, **f** FACS-isolated Th1, Th2, Th17 and Th1/17 cells were co-stimulated with DLL4/Cyt (Cyt = IFN-α/IL-6/IL-21) and treated with siRNA against c-Maf (si*MAF*) (**e**) or Blimp-1 (si*PRDM*1) (**f**) and control siRNA (siCtrl). Quantification of IL-10 production as measured by ELISA in cell culture supernatant. Each dot represents one healthy donor (*n* = 12–22). Two-tailed Wilcoxon and paired *t* tests were performed (**P < 0.01; ***P < 0.001) to assess significance. Data are cumulative from up to five independent experiments.

We next sought to elucidate the molecular networks underlying the induction of IL-10 in human memory CD4^+^ T cells. Therefore, we comparatively assessed the expression of several transcription factors, known to be involved in IL-10 regulation in mouse and humans^2^ in conventionally (TCR stimulation only) and DLL4 + STAT3 cytokine co-stimulated Th1, Th2, Th17 and Th1/17 cells via multiplex PCR (Fig. 4a). Interestingly, among all factors tested, the transcriptional regulators Blimp-1 (encoded by *PRDM1*) and c-Maf (encoded by *MAF*) were most highly overexpressed upon DLL4 + STAT3 cytokine co-stimulation in all Th cell subsets (Fig. 4a). Consistently, intracellular protein staining revealed strongly increased frequencies of Blimp-1- and c-Maf-expressing cells among memory CD4^+^ T cells co-stimulated with DLL4 + STAT3 cytokines (Fig. 4b). Furthermore, IL-10-expressing cells were significantly enriched among Blimp-1-positive or c-Maf-positive CD4^+^ T cells, respectively, as compared to their corresponding negative counterparts (Fig. 4c) and maximal IL-10 expression was detected in Blimp-1/c-Maf-double-positive cells (Fig. 4d). This suggests a key cooperative role of both transcription factors in mediating IL-10 expression.

In order to functionally test this hypothesis, we knocked-down Blimp-1 and c-Maf expression by siRNA treatment in DLL4 + STAT3 cytokine co-stimulated Th1, Th2, Th17 and Th1/17 cells (Fig S4a). Strikingly, si*PRDM1* or si*MAF* treatment almost completely abolished IL-10 production in all Th cell subsets (Fig. 4e, f). Importantly, the downregulation was specific to IL-10, as IFN-γ and/or IL17A production by Th1, Th17 or Th1/17 cells was completely unaffected by the treatment (Fig. S4b). Only Th2 cells also showed diminished IL-4 production upon si*PRDM1* or s*iMAF* treatment (Fig. S4b). To gain more insight into the functional relationship between Blimp-1 and c-Maf, we tested whether both factors controlled their mutual expression. However, neither the siRNA-mediated knock-down of *PRDM1* nor *MAF* resulted in a significant change in *MAF* or *PRDM1* expression, respectively (Fig. S4c), indicating that both factors contributed independently to IL-10 regulation.

In summary, these data showed that the acquisition of a regulatory phenotype by human memory CD4^+^ T cells upon Notch and STAT3 cytokine co-stimulation was accompanied by a specific upregulation of Blimp-1 and c-Maf, which jointly controlled IL-10 expression.

### Blood memory CD4^+^ T cells from IBD patients exhibit an inflammatory cytokine profile and disease-specific impairment of IL-10 production

In humans, polymorphisms in IL-10 and IL-10 receptor are associated with IBD, such as Crohn’s disease (CD) and ulcerative Colitis (UC).^3^ Similarly, rare loss-of-function mutations in IL-10 or IL-10 receptor cause severe, very early-onset IBD in infants and children.^3^ However, despite this essential role of IL-10 for the prevention of IBD, the role and possible deficits of IL-10 production by blood-derived effector memory CD4^+^ T cells in IBD have not been studied in detail.

**Fig. 5:**
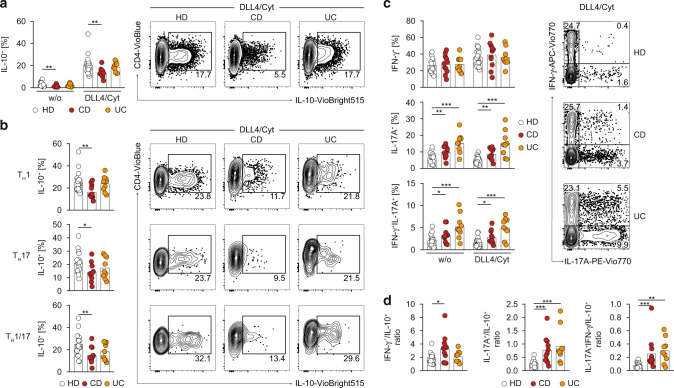
Notch/STAT3-mediated acquisition of IL-10 expression is defective in Crohn’s disease patients. **a** Effector memory CD4^+^ T cells were isolated from blood of healthy donors (HD), Crohn’s disease (CD) or ulcerative colitis (UC) patients. Afterwards, cells were cultured and co-stimulated w/ or w/o DLL4/Cyt (Cyt = IFN-α/IL-6/IL-21) for 120 h and restimulated with PMA/Ionomycin for optimal cytokine detection. **a** Quantification of IL-10^+^ cells (left) and representative flow cytometric profiles of CD4 vs. IL-10 expression after DLL4/Cyt (Cyt = IFN-α/IL-6/IL-21) co-stimulation (right). **b** Quantification of IL-10^+^ cells among Th1 (IFN-γ^+^), Th17 (IL-17A^+^) and Th1/17 (IFN-γ^+^IL-17A^+^) cells from HD, CD and UC patients after DLL4/Cyt (Cyt = IFN-α/IL-6/IL-21) costimulation (left). Representative flow cytometric profiles of CD4 vs. IL-10 expression after DLL4/Cyt (Cyt = IFN-α/IL-6/IL-21) co-stimulation (right). **c** Quantification of IFN-γ^+^, IL-17A^+^ and IFN-γ^+^IL-17A^+^ cells among effector memory CD4^+^ T cells from HD, CD and UC patients co-stimulated w/ or w/o DLL4/Cyt (Cyt = IFN-α/IL-6/IL-21) (left). Representative flow cytometric profiles of IFN-γ vs. IL-17A expression after DLL4/Cyt (Cyt = IFN-α/IL-6/IL-21) co-stimulation (right). **d** Ratio of IFN-γ^+^, IL-17A^+^ and IFN-γ^+^IL-17A^+^ cells to IL-10^+^ cells (frequency) among effector memory CD4^+^ T cells from HD, CD and UC patients co-stimulated w/ or w/o DLL4/Cyt (Cyt = IFN-α/IL-6/IL-21). Each dot represents one donor (*n*_HD_=19, *n*_CD_ = 11, *n*_UC_ = 9). Two-tailed Mann-Whitney and unpaired t tests were performed (* *P* < 0.05; ** *P* < 0.01; *** *P* < 0.001) to assess significance. Data are cumulative from nine independent experiments.

Therefore, we compared the expression of IL-10 between PBMC-derived memory CD4^+^ T cells from healthy donors (HD) and CD and UC patients. Indeed, as compared to HD, memory CD4^+^ T cells from CD patients produced less IL-10 both after TCR-only stimulation as well as after IL-10-promoting DLL4 + STAT3 cytokine co-stimulation (Fig. 5a). In contrast, memory CD4^+^ T cells from UC patients showed no overall reduction of IL-10 expression (Fig. 5a). Interestingly, a more detailed analysis of DLL4 + STAT3 cytokine-induced IL-10 production by individual Th cell subsets revealed a uniform reduction of IL-10 expression among Th1 (IFN-γ^+^), Th17 (IL-17^+^) and Th1/17 (IL-17^+^/IFN-γ^+^) cells as compared to HD (Fig. 5b). This reduction in individual subsets was significant for CD but not for UC patients despite a similar trend in the Th1/17 and Th17 cell subsets. Thus, consistent with our finding that DLL4 + STAT3 cytokine-induced IL-10 expression was independent of a specific Th cell differentiation program, impairment of memory CD4^+^ T cell- derived IL-10 expression in CD patients was not restricted to a specific Th cell subset.

In addition to changes in IL-10 expression, we also detected elevated frequencies of IL-17^+^ and IL-17^+^/IFN-γ^+^ cells in CD as well as in UC patients, whereas the frequency of IFN-γ single-producing cells was not altered as compared to HD (Fig. 5c). Consequently, these alterations in cytokine production resulted in a strong imbalance between pro- and anti-inflammatory cytokine expression in IBD, as evidenced by increased ratios of IFN-γ^+^, IL-17^+^ and IL-17^+^/IFN-γ^+^ to IL-10^+^ memory CD4^+^ T cells in CD and UC patients (Fig. 5d).

Thus, blood-derived memory CD4^+^ T cells from IBD patients exhibited a skewed pro-inflammatory over anti-inflammatory cytokine balance in response to Notch/STAT3 co-stimulation, which was characterized by reduced expression of IL-10 in CD patients and increased inflammatory IL-17 and IL-17/IFN-γ co-production in CD and UC patients.

### Impaired memory CD4^+^ T-cell-derived IL-10 production in CD patients is associated with reduced Blimp-1 and c-Maf expression

The defective acquisition of IL-10 production by memory CD4^+^ T cell from CD patients raised the question whether alterations in Blimp-1 and/or c-Maf expression were underlying this impairment.

**Fig. 6:**
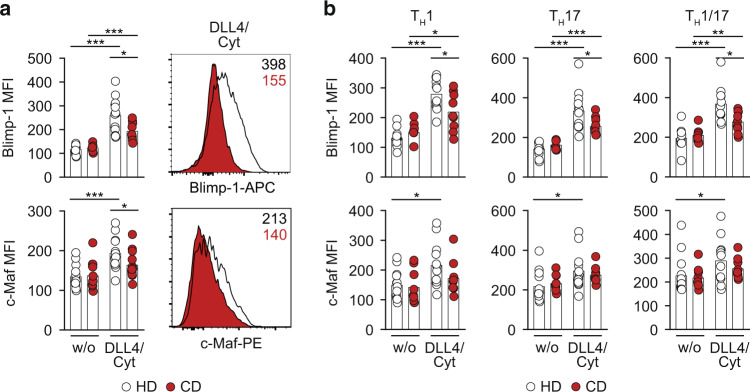
Impaired IL-10 expression in Crohn’s disease patients is associated with reduced Blimp-1/c-Maf expression. **a** Effector memory CD4^+^ T cells were isolated from blood of healthy donors (HD) or Crohn’s disease (CD) patients. Afterwards, cells were cultured and co-stimulated w/ or w/o DLL4/Cyt (Cyt = IFN-α/IL-6/IL-21) for 120 h. Quantification of Blimp-1 and c-Maf MFI among effector memory CD4^+^ T cells (left) and representative histograms (right). **b** Quantification of Blimp-1 and c-Maf MFI among Th1 (IFN-γ^+^), Th17 (IL-17A^+^) and Th1/17 (IFN-γ^+^IL-17A^+^) cells from HD and CD patients co-stimulated w/ or w/o DLL4/Cyt (Cyt = IFN-α/IL-6/IL-21). Each dot represents one donor (*n*_HD_=13, *n*_CD_ = 9). Two-tailed Mann–Whitney and unpaired *t* tests were performed (* *P* < 0.05; ** *P* < 0.01; *** *P* < 0.001) to assess significance. Data are cumulative from four independent experiments.

Therefore, we measured Blimp-1 and c-Maf protein expression by flow-cytometry in memory CD4^+^ T cells from HD and CD patients after TCR-only stimulation as well as after DLL4 + STAT3 cytokine co-stimulation. While there was no difference in Blimp-1 and c-Maf expression after conventional TCR stimulation, both factors were indeed significantly reduced in CD patients after IL-10-promoting DLL4 + STAT3 cytokine co-stimulation as compared to HD (Fig. 6a). In fact, memory CD4^+^ T cells from CD patients completely failed to upregulate c-Maf expression in response to DLL4 + STAT3 cytokine co-stimulation (Fig. 6a). Importantly, similar to the impaired IL-10 expression, the defective upregulation of Blimp-1 and c-Maf expression in CD patients was not restricted to a particular Th cell subset, but similarly detectable in Th1 (IFN-γ^+^), Th17 (IL-17^+^) and Th1/17 (IL-17^+^/IFN-γ^+^) cells (Fig. 6b). Notably, the altered response of memory CD4^+^ T cells from CD patients to DLL4 + STAT3 cytokine co-stimulation was not due to differences in Notch or cytokine receptor expression (Fig S5a, and S5b).

Taken together, these data provided a molecular explanation for the defective acquisition of self-regulatory IL-10 expression upon Notch/STAT3 co-stimulation by effector memory CD4^+^ T cells from CD patients as compared to healthy controls.

## Discussion

The acquisition of immunosuppressive IL-10 production by inflammatory effector memory CD4^+^ T cells is an essential autoregulatory mechanism to limit exaggerated inflammation and collateral tissue damage. Although advances have been made to unravel the molecular control of IL-10 in murine CD4^+^ T cells,^2,52,53^ the respective extra- and intracellular mediators governing IL-10 expression in human effector CD4^+^ T cells are less well understood. Here, we identified a Notch/STAT3-dependent signaling module to specifically induce IL-10 expression in human naïve and diverse effector memory CD4^+^ T cell subsets, including Th1, Th2, Th17 and Th1/17 cells. Downstream of Notch/STAT3, we identified the transcriptional regulators Blimp-1 and c-Maf to jointly control IL-10 expression, establishing a novel universal anti-inflammatory pathway in human CD4^+^ T cells. Importantly, our analysis of blood-derived effector CD4^+^ T cells from IBD patients revealed defects in Notch/STAT3-induced IL-10 production, which were associated with reduced Blimp-1 and c-Maf expression, suggesting that dysregulation of the Notch/STAT3-IL-10 axis in effector CD4^+^ T cells might contribute to disease development and/or progression.

The identification of Notch as a potent driver of human CD4^+^ T cell-derived IL-10 expression expands our previous work and that of others, which demonstrated an essential role of Notch for IL-10 expression by murine effector CD4^+^ T cells, predominantly Th1 cells, in vitro and in vivo.^32–35,54^ Interestingly, in humans, Notch signaling has been shown to intersect with the CD46-mediated IL-10 switching of Th1 cells,^55^ corroborating the key role of Notch for the control of IL-10 expression by murine and human effector CD4^+^ T cells. In mice, Notch synergized with STAT4 downstream of IL-12 or IL-27 to induce IL-10 in Th1 cells.^32,35^ In addition, also STAT1 and STAT3 (downstream of IL-21, IL-27 and IL-6, respectively),^20,22,23,26,41^ as well as STAT6 (downstream of IL-4),^17,56^ were shown to regulate murine CD4^+^ T cell-derived IL-10 production. Here, we found a dominant role of STAT3 downstream of IFN-α, IL-6 and IL-21 but not STAT1 and STAT4. In fact, siRNA-mediated knock-down of STAT1 downstream of IFN-α even enhanced IL-10 production (Fig. 1d), confirming the reciprocal STAT1/STAT3 regulatory relationship described in previous reports.^57–61^ Since Notch, via Hes-1 induction, is a known enhancer of STAT3 signaling in various cell types,^62–64^ enhancement of STAT3 over STAT1 activation downstream of IFN-α may explain the strong synergistic effect of IFN-α and Notch.

Our data also identified Blimp-1 and c-Maf as cooperative transcriptional regulators of IL-10 in human CD4^+^ T cells. Both factors act as master regulators of IL-10 in various murine CD4^+^ T cell subsets, where they drive IL-10 expression by directly binding and transactivating the *Il10* gene.^27,35^ Two recent studies could link c-Maf also to the regulation of IL-10 in human Th1 and Th17 cells, respectively,^44,45^ and demonstrate c-Maf binding to human *IL10* as well.^44^ Mechanistically, our data suggest synergistic roles of Blimp-1 and c-Maf in IL-10 regulation, since the knock-down of each factor was sufficient to drastically reduce IL-10 expression. This is consistent with murine Blimp-1/c-Maf double knock-out and overexpression approaches, which have established complementary but indispensable roles of both transcription factors in IL-10 regulation.^27,35^ In mice, Notch is mainly potentiating cytokine-driven c-Maf rather than Blimp-1 expression.^35^ Thus, although not directly tested here, we hypothesize that both factors are induced by STAT3 signaling in human CD4^+^ T cells, while c-Maf expression may be further enhanced by Notch activation. Obviously, the Blimp-1/c-Maf transcriptional module identified here does not exclude the contribution of other transcription factors, such as Eomes, which has previously been associated with the development of murine and human Tr1 cells.^65–67^

It is currently discussed, whether CD4^+^ T cells can differentiate into a stable IL-10-secreting CD4^+^ T cell subset or whether different effector CD4^+^ T cell subsets can dynamically switch on IL-10 as a common inducible property.^10,68^ Both scenarios are not mutually exclusive. Our data show that Notch/STAT3 co-stimulation induced Blimp-1/c-Maf-dependent IL-10 expression in all major CD4^+^ T cell subsets (naïve, Th1, Th2, Th17, Th1/17) independently of their polarization status and effector cytokine expression and without major effects on the expression of subset-defining effector cytokines. Following one round of stimulation the capacity of CD4^+^ T cells to co-express IL-10 in response to Notch/STAT3 was rapidly lost in the absence of these co-stimulators, as previously reported for different murine and human effector CD4^+^ T cells.^18,44^ Thus it remains to be shown, under which conditions and by which molecular mechanisms IL-10 expression is stabilized in established T helper subsets and whether this is accompanied by loss of effector cytokine expression, which are essential criteria for their therapeutic application.

In this context, it was interesting that also the expression of several co-inhibitory receptors was potently induced by Notch/STAT3. This included co-expression of LAG-3 and CD49b, a well-established combination of markers for the ex vivo identification of murine and human IL-10-producing Tr1 cells.^51^ We also found other co-inhibitory receptors, such as PD1, recently identified to be expressed on human intestinal Tr1 cells,^69^ TIM-3 and TIGIT to be enhanced on Notch/STAT3- co-stimulated CD4^+^ T cells. Interestingly, co-expression of co-inhibitory receptors, such as LAG-3, CD49b, PD-1, TIM-3 and TIGIT by IL-10-producing Tr1 cells correlates with their suppressive capacity.^70^ Thus Notch/STAT3 signals appear to regulate multiple molecular components of the Tr1 cell signature.^10^ On the transcriptional level, a recent study identified Blimp-1 and c-Maf as global regulators of a co-inhibitory receptor expression program in murine CD4^+^ T cells,^71^ and our data suggest a similar role for Blimp-1/c-Maf in human CD4^+^ T cells. However, as discussed above the in vitro co-stimulation with Notch/STAT3 did not induce a stable Tr1 cell phenotype.^10^ In particular, IL-10 production was transient and we did not detect a downregulation of effector cytokine production, which is typically seen in Tr1 or exhausted T cells.^10^ Thus, it is possible that additional external signals, such as repetitive or chronic stimulation, are needed to stabilize the Notch/STAT3-dependent immunoregulatory program.

Finally, we interrogated the Notch/STAT3-dependent IL-10-inducing pathway in effector memory CD4^+^ T cells from IBD patients. Indeed, genome-wide association studies have shown that several nodes of this pathway, such as *IFNAR*, *STAT3*, *PRDM1* and *IL10* itself, represent risk loci for IBD development.^3,72–74^ In support of these genomic associations, we found that blood-derived effector memory CD4^+^ T cells from CD patients exhibited impaired IL-10 production upon Notch/STAT3 co-stimulation, which was associated with diminished Blimp-1 and c-Maf expression. Interestingly, this impairment in IL-10 production was not present in UC patients, suggesting a disease-specific role of memory CD4^+^ T cell-derived IL-10 in IBD. Nevertheless, we found that effector memory CD4^+^ T cells from both CD and UC patients overexpressed inflammatory IL-17 and IL-17/IFN-γ, thus representing a hallmark of IBD.^75–77^ Importantly, our data are consistent with recent studies, reporting reduced IL-10 expression from intestinal IFN-γ-producing Tr1 cells, reduced LAG-3^+^CD49b^+^IL-10^+^ Tr1 cells or dysregulation of the intestinal Type I interferon-IL-10 axis in patients with IBD.^69,70,78^ Of note, expression of Notch- and cytokine receptors by naïve and memory CD4^+^ T cells was neither affected by disease status nor the culture conditions, suggesting that the IL-10/c-Maf/Blimp-1 dysregulation occurs further downstream of these inducing signaling pathways. It will be important to address the molecular regulation of *PRDM1* and *MAF* expression including the impact of genetic diversity.

Collectively, these findings suggest that imbalanced expression of pro-inflammatory cytokines and immunosuppressive IL-10 by human effector memory CD4^+^ T cells critically contributes to IBD development and/or progression. The identification of the Notch/STAT3 – Blimp-1/c-Maf axis as a universal pathway regulating anti-inflammatory functions in human CD4^+^ T cells may represent novel therapeutic targets for the treatment of intestinal chronic inflammatory diseases.

## Materials and methods

### Human samples

Buffy coats, peripheral EDTA or heparin blood samples from healthy donors were obtained from the Charité Universitätsmedizin Berlin blood bank, or from local healthy volunteers. Frozen PBMC or peripheral EDTA blood samples from patients with Crohn’s disease (CD) or ulcerative colitis (UC) were obtained from Charité Universitätsmedizin Berlin, Campus Benjamin Franklin, Department of Gastroenterology, Infectious Diseases and Rheumatology. Age, sex, C-reactive protein, disease activity scores (Harvey-Bradshaw-Index for CD and Mayo Score for UC), medication and disease duration were documented on the day of sample collection (Table S1). All clinical investigation was conducted according to the Declaration of Helsinki principles. All blood donors gave informed consent (ethics committee Charité, EA4/094/12).

### PBMC isolation

For isolation of peripheral blood mononuclear cells (PBMC), blood samples were overlaid to buffered Biocoll separating solution, density 1077 g/ml (Biochrom, Berlin, Germany) and density gradient centrifugation was performed at 1000 × g for 30 min at room temperature (RT) without brakes.

### FACS isolation

For isolation of human naïve and memory CD4^+^ T cells, CD4^+^ cells were enriched from PBMC via MACS using CD4 Microbeads (Miltenyi Biotec, Bergisch Gladbach, Germany) according to the manufacturer’s instructions. Subsequently, MACS-enriched CD4^+^ T cells were further purified via FACS. Therefore, 5 × 10^7^ MACS-enriched CD4^+^ T cells were stained with fluorochrome labeled antibodies in 100 µl PEB buffer (1x PBS, 2 mM EDTA, 0, 5% FBS) for 10 min at 4 °C in the dark. Naïve and memory CD4^+^ T cells were defined as CD4^+^CD45RA^+^CD45R0^-^CCR7^+^CD31^+^ and CD4^+^CD45RA^−^CD45R0^+^CD31^−^, respectively. For discrimination of T_H_ subsets, MACS-enriched CD4^+^ T cells were stained for CD4, CD45R0, CCR10, CCR4, CCR6, CXCR3 and isolated according to their subset-specific chemokine receptor expression pattern.^47^ T_H_1 cells: CD4^+^CD45R0^+^CXCR3^+^CCR4^−^CCR6^+^, T_H_2 cells: CD4^+^CD45R0^+^CCR4^+^CXCR3^–^CCR6^–^, T_H_17 cells: CD4^+^CD45R0^+^CCR6^+^CCR4^+^CXCR3^–^ and T_H_1/17 cells: CD4^+^CD45R0^+^CCR6^+^CXCR3^+^CCR4^–^ (Fig. S2). For isolation of memory CD4^+^ T cells (CD3^+^CD4^+^CD45R0^+^) from CD and UC patients and corresponding healthy donors, PBMC were stained for CD4 and CD45R0 and FACS-purified without previous MACS enrichment. The following antibodies were used in this study: CCR10-PE (REA326, 1:10), CCR4-PerCP-Cy5.5 (L291H4, 1:20, Biolegend, San Diego, CA, USA), CCR6-PE-Vio770 (REA190, 1:10), CCR7-PE-Vio770 (REA108, 1:10), CD3-VioBlue (BW264/56, 1:50), CD31-APC (AC128, 1:20), CD31-VioBlue (AC128, 1:20), CD4-APC-Vio770 (VIT4, 1:50), CD45RA-FITC (T6D11, 1:40), CD45R0-PE (UCHL1, 1:160), CD45R0-FITC (UCHL1, 1:20), CD45R0-PerCP (UCHL1, 1:20), CXCR3-APC (REA232, 1:10). If not mentioned otherwise, all antibodies were purchased from Miltenyi Biotec. FACS isolation was performed on FACSAria^TM^ or FACSAria^TM^II (BD Biosciences, Franklin Lakes, NJ, USA).

### Cell culture

In 96-well cell plates, 5 × 10^4^ CD4^+^ T cells/well were cultured in 200 µl DMEM + GlutaMAX™ (Thermo Fisher Scientific, Waltham, MA, USA), supplemented with 100 U/ml penicillin-streptomycin (Thermo Fisher Scientific), 50 µg/ml gentamycin (PAN Biotech, Aidenbach, Germany), 10% (v/v) heat-inactivated fetal bovine serum (Biowest, Riverside, MO, USA) and 50 μM 2-Mercaptoethanol (PAN Biotech) in the presence of 5 × 10^4^ CD3-Biotin/CD28-Biotin/CD2-Biotin labeled anti-Biotin MACSiBead^TM^ particles and 400 U/ml IL-2 (Proleukin®, Novartis Pharma, Nuremberg, Germany) for 120 h, following a 24 h resting phase by replacement of the cell culture media with 200 µl new DMEM without any cytokines. Notch activation via DLL4 was induced by addition of 15 × 10^4^ MACSi Beads/well, covalently coated with recombinant mouse DLL4 (rmDLL4-hFc, kindly provided by Antonius Rohlink, Basel, Switzerland) from transfected CHO cells. If indicated, cytokines IFN-α, IL-6, IL-21 were added to the cell culture medium at 20 ng/ml. If not mentioned otherwise, all reagents were purchased from Miltenyi Biotec.

### Flow cytometry

For surface staining of co-inhibitory receptors, cells were harvested after 48 h of cell culture. Cells were resuspended in 30 µl PEB containing fluorochrome labeled antibodies and stained for 10 min at 4 °C in the dark. Cells were washed and resuspended in PEB for acquisition. For intracellular staining cells were rested for 24 h after 120 h cell culture and then restimulated with Phorbol-12-myristat-13-acetat (PMA – 10 ng/ml, Sigma-Aldrich, St. Louis, MO, USA) and ionomycin (1 µg/ml, Sigma-Aldrich) for 5 h and after 1 h of incubation Brefeldin (5 µg/ml, Sigma-Aldrich) was added. Subsequently, cells were resuspended in 1x PBS containing Viobility^TM^ 405/520 Fixable Dye (1:5000) and stained for 15 min at RT in the dark to discriminate dead cells. Cells were then fixed and permeabilized with Inside Stain Kit according to manufacturer’s instructions. Cells were then resuspended in 30 µl Inside Perm solution containing fluorochrome labeled antibodies and stained for 20 min at RT in the dark. Cells were washed and resuspended in PEB for acquisition. For intranuclear staining, cells were resuspended in 100 µl Fix/Perm buffer from Foxp3 staining buffer set and incubated for 30 min at 4 °C in the dark. Subsequently, cells were washed in 1x Perm buffer and resuspended in 1x Perm buffer containing fluorochrome labeled antibodies and stained for 30 min at 4 °C in the dark. If surface, intracellular or intranuclear stainings were combined, protocols were performed subsequently. Data was acquired on a MACSQuant Analyzer 10 and MACSQuantify or FlowJo (Treestar, Ashland, OR, USA). Surface antibodies: CD4-VioBlue (VIT4, 1:50), CD49b-PE-Vio770 (REA188, 1:10), LAG-3-PE (3DS223H, 1:3,5, R&D Systems, Minneapolis, MN, USA), PD-1-APC (PD1.3.1.3, 1:10), TIGIT-Biotin (4E1.2, 1:10) Biotin-PerCP (REA746, 1:10), TIM3-VioBright-FITC (F38-2E2, 1:10). Intracellular antibodies: IFN-γ-APC-Vio770 (45-15, 1 :100), IFN-γ-PerCP-Cy5.5 (4 S.B3, 1 :160, Biolegend) IL-10-APC (JES3-9D7, 1 :50), IL-10-VioBright515 (JES3-9D7, 1 :50), IL-17A-APC-Vio770 (CZ8-23G1, 1 :50), IL-17A-PE-Vio770 (CZ8-23G1, 1 :50), IL-4-FITC (7A3-3, 1 :50), TNF-α-PEVio770 (cA2, 1 :50). Intranuclear antibodies: Blimp-1-APC (MAB36081, 1:20, R&D Systems), c-Maf-PE (sym0F1, 1:20, eBioscience Inc, San Diego, CA, USA). If not mentioned otherwise, all reagents, devices and software for flow cytometry were purchased from Miltenyi Biotec.

### High-throughput microfluidic real-time PCR

Memory CD4^+^ T cells were activated under the indicated conditions for 24 h and then 800 cells were resuspended in 2X Reaction Mix (from Super Script® III One-Step RT-PCR System) plus SUPERase-In^TM^ RNase Inhibitor and stored at −80 °C. Reverse transcription of specific RNA targets was performed using TaqMan Gene Expression Assays (Table S2) and SuperScript® III RT/Platinum® Taq Mix (from Super Script® III One-Step RT-PCR System). Subsequently, high throughput real-time PCR was performed using 48.48 Sample/Loading Kit in a Biomark HD (both purchased from Fluidigm, San Francisco, CA, USA) according to manufacturer’s instructions. If not mentioned otherwise, all reagents and devices from Thermo Fisher Scientific.

### siRNA treatment

For knockdown of *MAF* and *PRDM1* memory CD4^+^ T cells were activated in the presence of IFN-α, IL-6, IL-21 and DLL4 for 48 h. MACSiBeads were removed using MACSiMAG (Miltenyi Biotec) according to the manufacturer’s instructions. Subsequently, up to 5 × 10^5^ CD4^+^ T cells were rested for 2 h in RPMI 1640 (Thermo Fisher Scientific), supplemented with 5% human AB serum (Sigma-Aldrich), before transfection with 300 pmol siCtrl, siMAF, siPRDM1 respectively (Table S3) using P3 Primary Cell 96-well Nucleofector^TM^ Kit according to manufacturer’s instructions. Nucleofection was performed in 96-well Shuttle™ device of the Nucleofector™ 2b device, using nucleofection program ED-108. Subsequently, CD4^+^ T cells were rested in RPMI 1640 + 5% AB serum overnight. All reagents and devices for transfection were purchased from Lonza Group AG, Basel, Switzerland. Subsequently, 1 × 10^4^ CD4^+^ T cells were re-activated, using 1 × 10^4^ CD3-Biotin/CD28-Biotin/CD2-Biotin labeled Anti-Biotin MACSiBeads (Miltenyi Biotec) for additional 48 h before supernatants for cytokine detection were harvested. For nucleofection with si*STAT*1, si*STAT*3 and si*STAT*4 (Table S3) naïve T cells were used ex vivo for nucleofection with Human T Cell Nucleofector^TM^ Kit (Lonza). Up to 5 × 10^6^ cells were nucleofected with up to 1 nmol of siRNA in a Nucleofector™ 2b device (Lonza) according to manufacturer’s instructions using program X-001. Subsequently, cells were rested in RPMI + 5% AB serum for 24 h and used for cell culture.

### Detection of cytokines in cell culture supernatant

Supernatant of siRNA-transfected T cells was harvested 48 h after TCR restimulation stored at −20 °C. Cytokine levels were determined by ELISA using Ready-Set-Go!® ELISA for IL-10, IFN-γ, IL-4 or IL-17A (eBioscience Inc.) or with MACSPlex Cytokine 12 Kit, human (Miltenyi Biotec) according to manufacturer’s instructions, respectively. Data of MACSPlex Cytokine 12 Kit, human was acquired on a MACSQuant Analyzer 10 and MACSQuantify (both Miltenyi Biotec).

### Real-time PCR

T cell RNA isolation was performed with the RNeasy micro kit (Qiagen) following the manufacturer’s instructions and cDNA prepared using the RevertAid First Strand cDNA Synthesis Kit (Thermo Fisher Scientific) according to the manufacturer’s instructions. The resulting cDNA was amplified using the SYBR Select Master Mix and detected on a QuantStudio 5 Real-Time PCR System (Applied Biosystems). On completion of the PCR amplification, a DNA melting curve analysis was carried out to confirm the presence of a single amplicon. *ACTB* was used as an internal reference gene in order to normalize the transcript levels. Primers used are listed in (Table S4). Relative mRNA levels (2-ΔΔCt) were determined by comparing the PCR cycle thresholds (Ct) for the gene of interest and internal reference gene (ΔCt) and ΔCt values for treated and control groups (ΔΔCt).

### Suppression assay

CD14^+^ cells were isolated from PBMC by positive selection using CD14 human microbeads (Miltenyi Biotec) according to manufacturer’s instructions. The cells were cultured (5 × 10^5^ cells/mL) in DMEM L-Glutamine medium containing 10% heat-inactivated fetal calf serum and supplemented with 50 µM 2-Mercaptoethanol and 250 µM recombinant human IL-2 using flat-bottom 96-well suspension plates. Cells were stimulated with 1 ng/ml ultrapure LPS (Invivogen, San Diego, CA, USA) in the presence or absence of conditioned media (1:50 dilution) from T cells cultured with CD3/CD28 +/− DLL4/STAT3 cytokines. Anti-IL10R blocking antibody (clone 3F9) was added where indicated (1 µg/ml). After 7 h, cells were collected and gene expression was analysed via Real-Time PCR.

### Statistical analysis

Statistical testing was performed with GraphPad Prism 6.0 (GraphPad, La Jolla, CA, USA). Data sets were tested for normality using the D’Agostino and Pearson omnibus normality test, followed by statistical significance testing using the appropriate statistical tests as indicated in the legends.

## Supplementary information

Supplementary Materials
