# Supplementary material for: A Notch/STAT3-driven Blimp-1/c-Maf-dependent molecular switch induces IL-10 expression in human CD4+ T cells and is defective in Crohn´s disease patients
Source: Mucosal Immunol. 2022 Feb 15;15(3):480–90. doi: 10.1038/s41385-022-00487-x (PMC9038525; doi:10.1038/s41385-022-00487-x)

Fig. S1 siRNA-mediated knock-down of STAT1, STAT3, STAT4 in human effector memory CD4$^+$ T cells. FACS-isolated effector memory CD4$^+$ T cells were co-stimulated with DLL4/Cyt (Cyt = IFN-α/IL-6/IL-21) and treated with siRNA against STAT1 (si*STAT1*), STAT3 (si*STAT3*), STAT4 (si*STAT4)* and control siRNA (siCtrl). Relative expression of *STAT1, STAT3, STAT4* after siRNA treatment, normalized to siCtrl treatment (set to 1).

Fig. S2 FACS isolation of human blood-derived CD4$^+$ Th cell subsets. a) Representative flow cytometric staining of various CD4$^+$ Th cell subsets (Th1, Th2, Th17, Th1/17) according to their chemokine receptor expression [47]. **b)** Expression of IFN-γ, IL-4 and IL-17 by FACS-isolated CD4$^+$ Th cell subsets. Cells were either stimulated with PMA/Ionomcyin *ex vivo* and stained intracellularly for indicated effector cytokines (upper panel) or cultivated for 120h to measure cytokines in cell culture supernatants by ELISA. Each dot represents on healthy donor (n=5-14). Data are cumulative from up to four independent experiments.

Fig. S3 Monocyte suppression assay. MACS separated CD14+ blood monocytes were activated with LPS in the presence or absence of cell culture supernatants (dilution 1:50) from memory CD4$^+$ T cells activated for 48h with CD3/CD28 alone (sup w/o) or plus DLL4/STAT3 (sup Dll4/Cyt). To the latter culture also anti-IL-10 receptor antibody was added. Relative *CD40* and *HLA-DR* expression after 6 hours of activation was analyzed via RT-PCR. Experiments with supernatants from three independent CD4$^+$ T cell cultures are shown.

Fig. S4 siRNA-mediated knock-down of Blimp-1 and c-Maf in human effector memory CD4$^+$ Th cell subsets. a) FACS-isolated Th1, Th2, Th17 and Th1/17 cells were co-stimulated with DLL4/Cyt (Cyt = IFN-α/IL-6/IL-21) and treated with siRNA against c-Maf (si*MAF*) or Blimp-1 (si*PRDM*1) and control siRNA (siCtrl). Relative expression of *MAF* and *PRDM*1 after si*MAF* or si*PRDM*1 treatment, respectively, normalized to siCtrl treatment (set to 1). **b)** Expression of IFN-γ, IL-4 and IL-17 by CD4$^+$ Th cell subsets as measured by ELISA in cell culture supernatants after treatment with si*MAF* or si*PRDM*1. **c)** Relative expression of *PRDM*1 and *MAF* after si*MAF* or si*PRDM*1 treatment, respectively, normalized to siCtrl treatment (set to 1).

Fig. S5 Expression of Notch and cytokine receptors by T cells from IBD patient and healthy controls. Naïve and memory CD4 T cells from CD, UC and healthy controls (each n= 7-10) were FACS-sorted and activated for 48 hours with CD3/CD28. Relative expression of Notch-1-4, and cytokine receptors for type I IFN, IL-6 and IL-21 were analyzed via RT-PCR.

Tab. S1 Patient characteristics

Patient	Underlying disease	Age	Sex	Harvey-Bradshaw-Index	Mayo Score	C-Reactive protein (mg/L)	Medication	Disease duration (year)
1	Ulcerative colitis	40	m		3	<1	Infliximab	20
2	Ulcerative colitis	27	m		1	<1	Prednisolon, Budesonid	7
3	Crohn's disease	40	w	5			Budesonid	10
4	Ulcerative colitis	22	m		1	<1	Thiopurine	5
5	Ulcerative colitis	24	m		0	<1	Vedolizumab	3
6	Crohn's disease	45	m	3		14,6	Thiopurine, Infliximab	21
7	Ulcerative colitis	44	m		5	1,2	Vedolizumab	6
8	Crohn's disease	64	w	0		<1,0	Thiopurine, Infliximab	16
9	Crohn's disease	56	w	1		1,2	Infliximab	n.a.
10	Crohn's disease	30	w	10		<1,0	Thiopurine, Infliximab	12
11	Ulcerative colitis	35	w		0	4,3	Infliximab	8
12	Crohn's disease	32	w	0		<1,0	Infliximab	13
13	Crohn's disease	50	w	3		3,9	Infliximab	1
14	Crohn's disease	53	w	3		19,3	Infliximab	13
15	Crohn's disease	55	w	15		2,6	Infliximab	16
16	Ulcerative colitis	35	w		0	1,1	Infliximab	1
17	Crohn's disease	49	m	1		6,5	none	1
18	Ulcerative colitis	41	w		2	<1	Infliximab	11
19	Crohn's disease	66	w	2		<1	Adalimumab	30
20	Ulcerative colitis	42	m		0	<1	5-aminosalicylic acid, E.coli Nissle	n.a.

Tab. S2 TaqMan® Gene Expression Assays

Gen	Assay#
AHR	Hs00907314_m1
BACH2	Hs00222364_m1
BATF	Hs00232390_m1
EGR2	Hs00166165_m1
GAPDH	Hs99999905_m1
GATA3	Hs00231122_m1
IKFZ3	Hs04329617_m1
IL10	Hs00961622_m1
IRF4	Hs00180031_m1
MAF	Hs04185012_s1
NFIL3	Hs00705412_s1
PRDM1	Hs00153357_m1

Tab. S3 siRNA sequences

siRNA	sequence	
siCtrl	sense	5' A AUU CUC CGA ACG UGU CAC GTT T 3'
	antisense	3' T TAA GAG GCU UGC ACA GUG CA 5'
si*MAF*	sense	5' A AAC GGC UCG AGC AGC GAC AAC C 3'
	antisense	3' T TUG CCG AGC UCG UCG CUG UT 5'
si*PRDM1*	sense	5' G ACG GCU UUA AUG AAG AGA AAA G 3'
	antisense	3' C TGC CGA AAU UAC UUC UCU UT 5'
si*STAT1*	sense	5’ ACA GAA AGA GCU UGA CAG TAA AG 3'
	antisense	3’ UAU CUU UCU CGA ACU GUC AUT 5’
si*STAT3*	sense	5’ GCG GAG AAG CAU CGU GAG TGA GC 3'
	antisense	3’ CGC CUC UUC GUA GCA CUC ACT 5’
si*STAT4*	sense	5’ AAA GAC AAA GCC UUC GGT AAA CA 3'
	antisense	3’ TTU CUG UUU CGG AAG CCA UUT 5’

Tab. S4 Real-Time PCR Primers

target	sequence	
ACTB	Forward 5'-3'	GAG CAC AGA GCC TCG CCT TT
	Reverse 5'-3'	TCA TCA TCC ATG GTG AGC TGG
IFNAR	Forward 5'-3'	AAA GCC AGA GCA CAC ACC AT
	Reverse 5'-3'	TGA CAA ACG GGA GAG CAA AT
IL6R	Forward 5'-3'	CTC AGT GTC ACC TGG CAA GAC
	Reverse 5'-3'	TCC TTG ACC ATC CAT GTT GTG
IL21R	Forward 5'-3'	GGG CTC TGT GAT GTA GGC AG
	Reverse 5'-3'	CCGT CTG GAG GTA ATC GGT G
NOTCH1	Forward 5'-3'	GGT GAA CTG CTC TGA GGA GAT C
	Reverse 5'-3'	GGA TTG CAG TCG TCC ACG TTG A
NOTCH2	Forward 5'-3'	GTG CCT ATG TCC ATC TGG ATG G
	Reverse 5'-3'	AGA CAC CTG AGT GCT GGC ACA A
NOTCH3	Forward 5'-3'	TAC TGG TAG CCA CTG TGA GCA G
	Reverse 5'-3'	CAG TTA TCA CCA TTG TAG CCA GG
NOTCH4	Forward 5'-3'	TTC CAC TGT CCT CCT GCC AGA A
	Reverse 5'-3'	TGG CAC AGG CTG CCT TGG AAT C
PRDM1	Forward 5'-3'	AGA GGT TAT TGG AGT GAT GAG
	Reverse 5'-3'	GTT CTT AGG AAC TGT GTC ATT G
MAF	Forward 5'-3'	AAG TCG ACC ACC TCA AGC AG
	Reverse 5'-3'	AAT GTG GCG TAT CCC ACT GA
CD40	Forward 5'-3'	GCA GGC ACA AAC AAG ACT GA
	Reverse 5'-3'	ATA AAG ACC AGC ACC AAG AGG
HLA-DR	Forward 5'-3'	CAA CAG AGC GCC CAA GAA GA
	Reverse 5'-3'	CTC GCC TGA TTG GTC AGG ATT

Supplemenent Fig. 1 Ahlers et al.

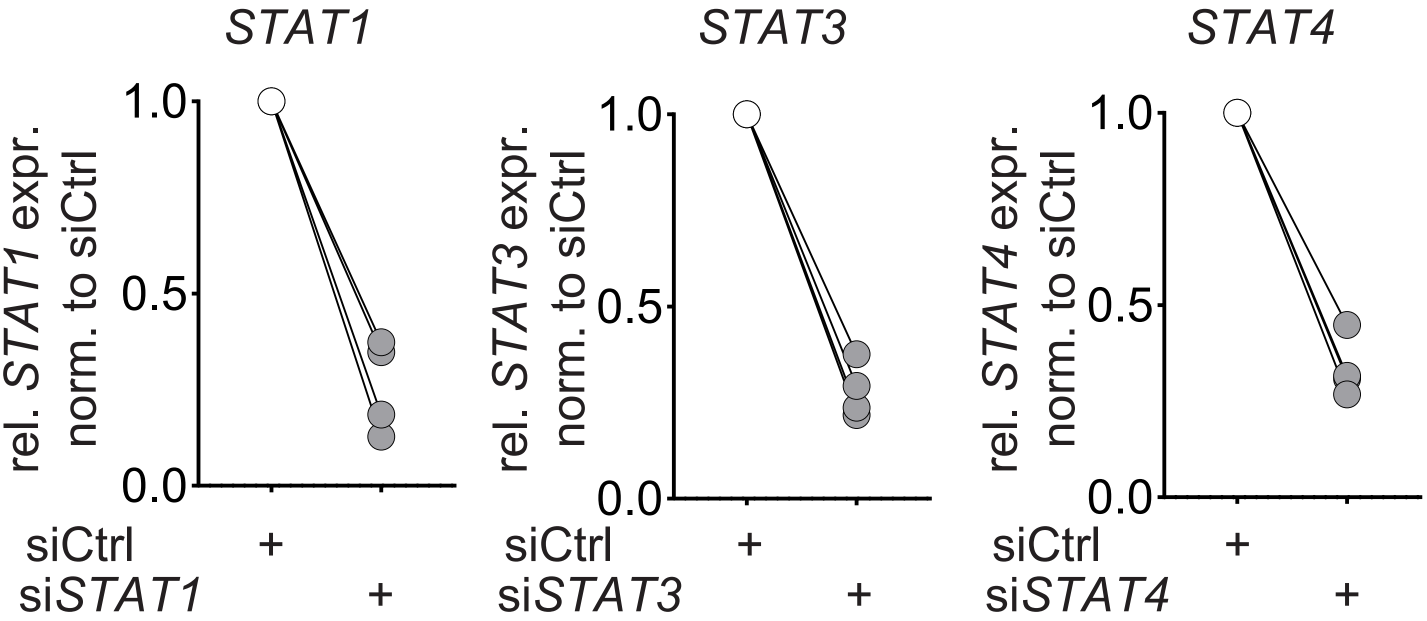

Supplement Fig. 2 Ahlers et al.

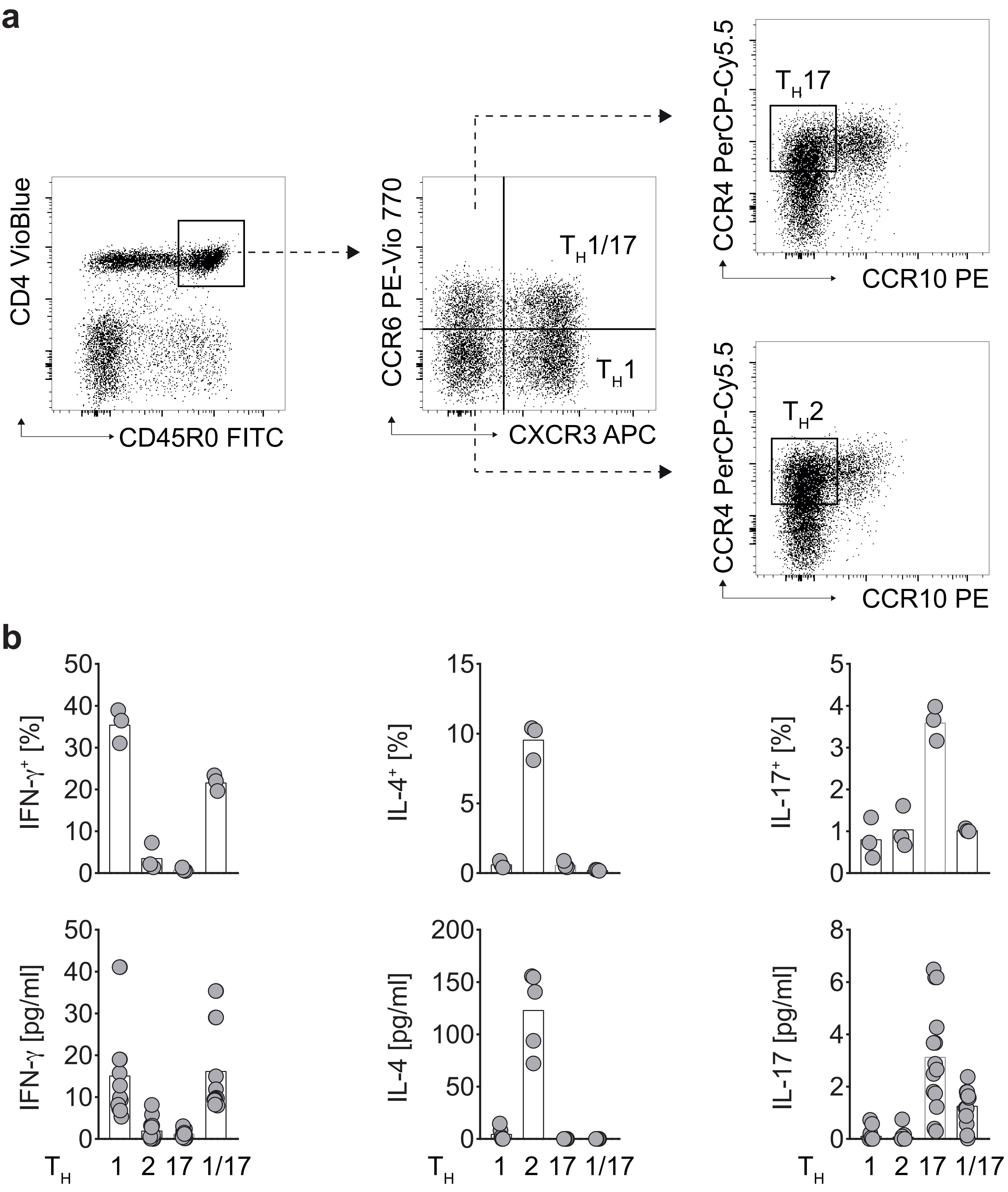

Supplement Fig. 3 Ahlers et al.

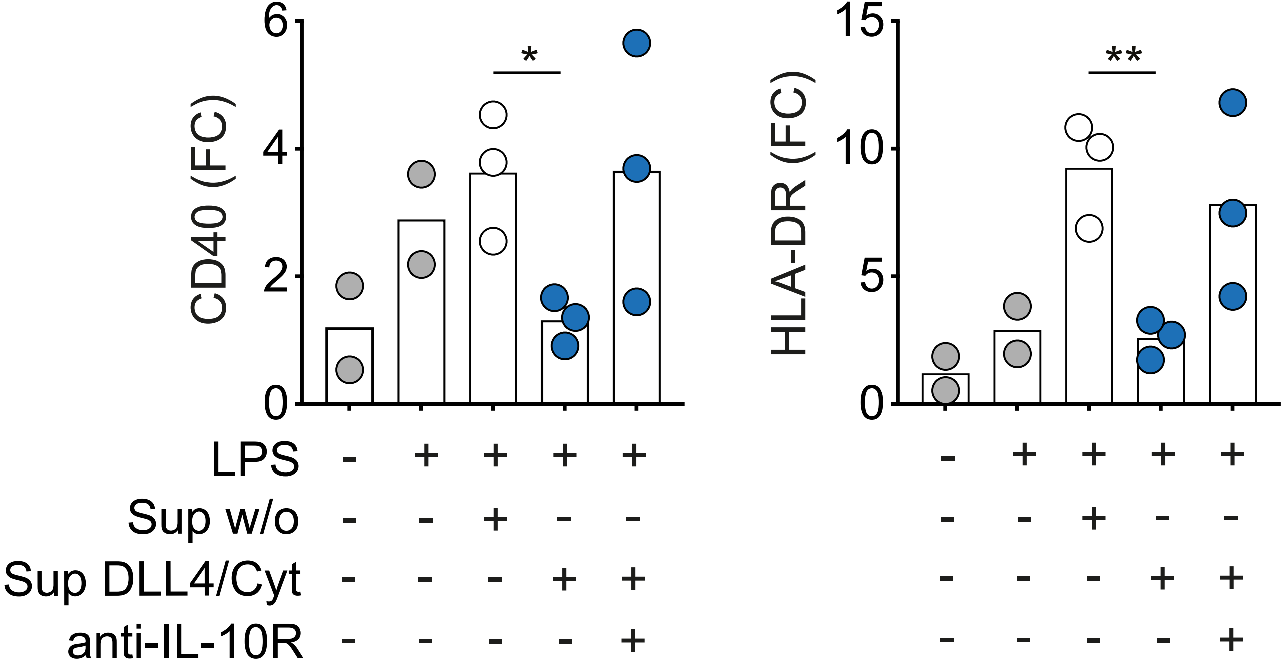

Supplement Fig. 4 Ahlers et al.

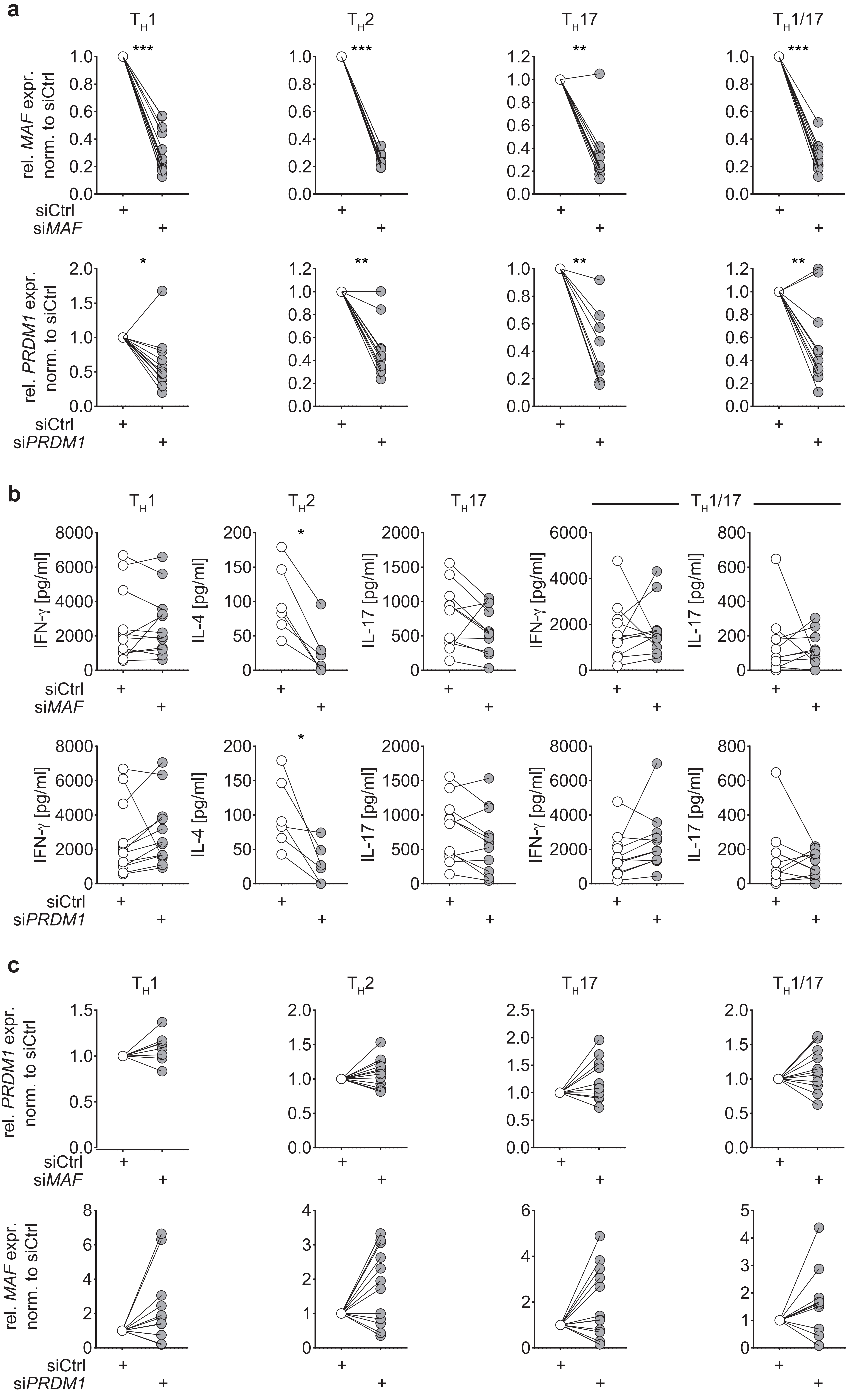

Supplement Fig. 5 Ahlers et al.

a

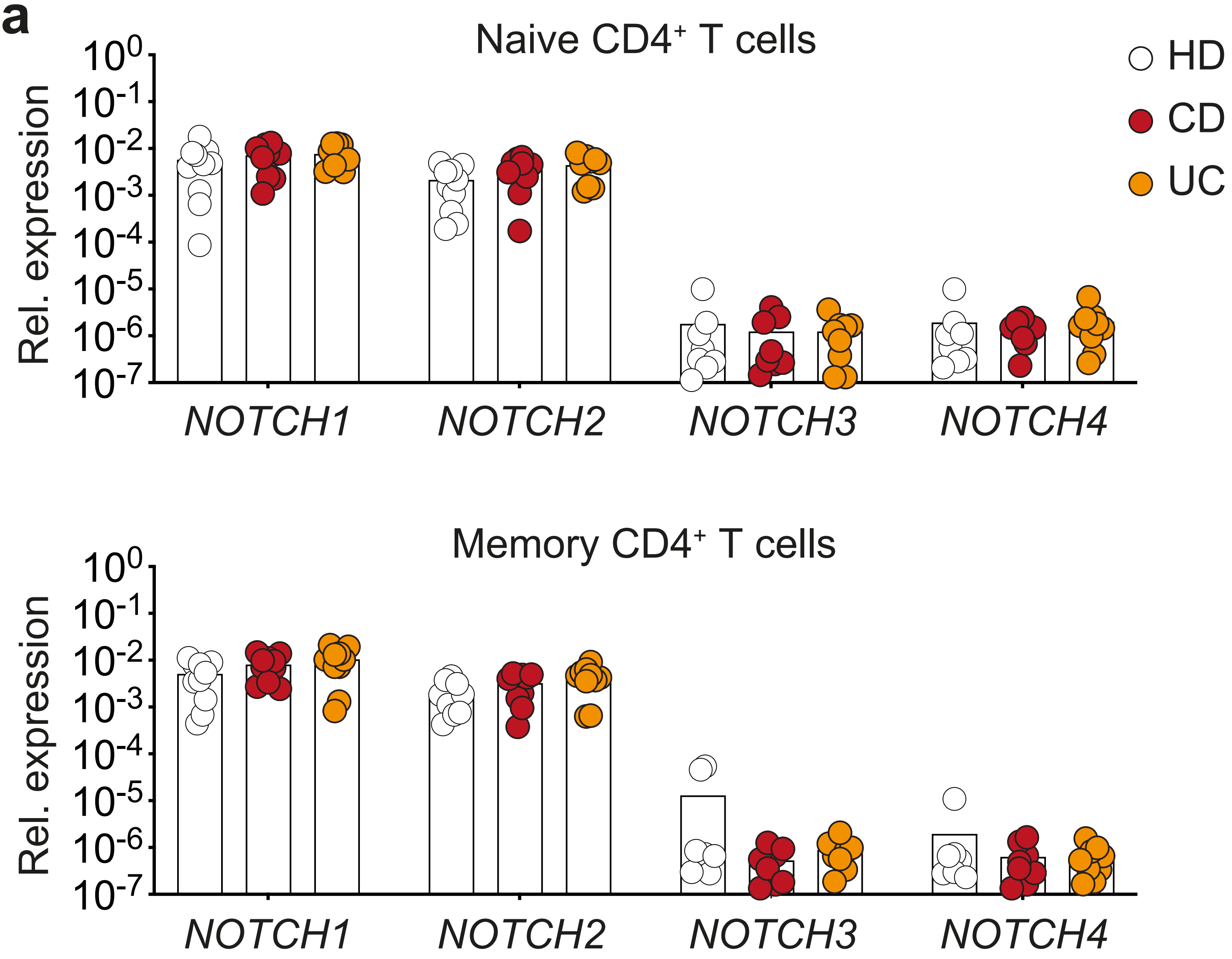

b

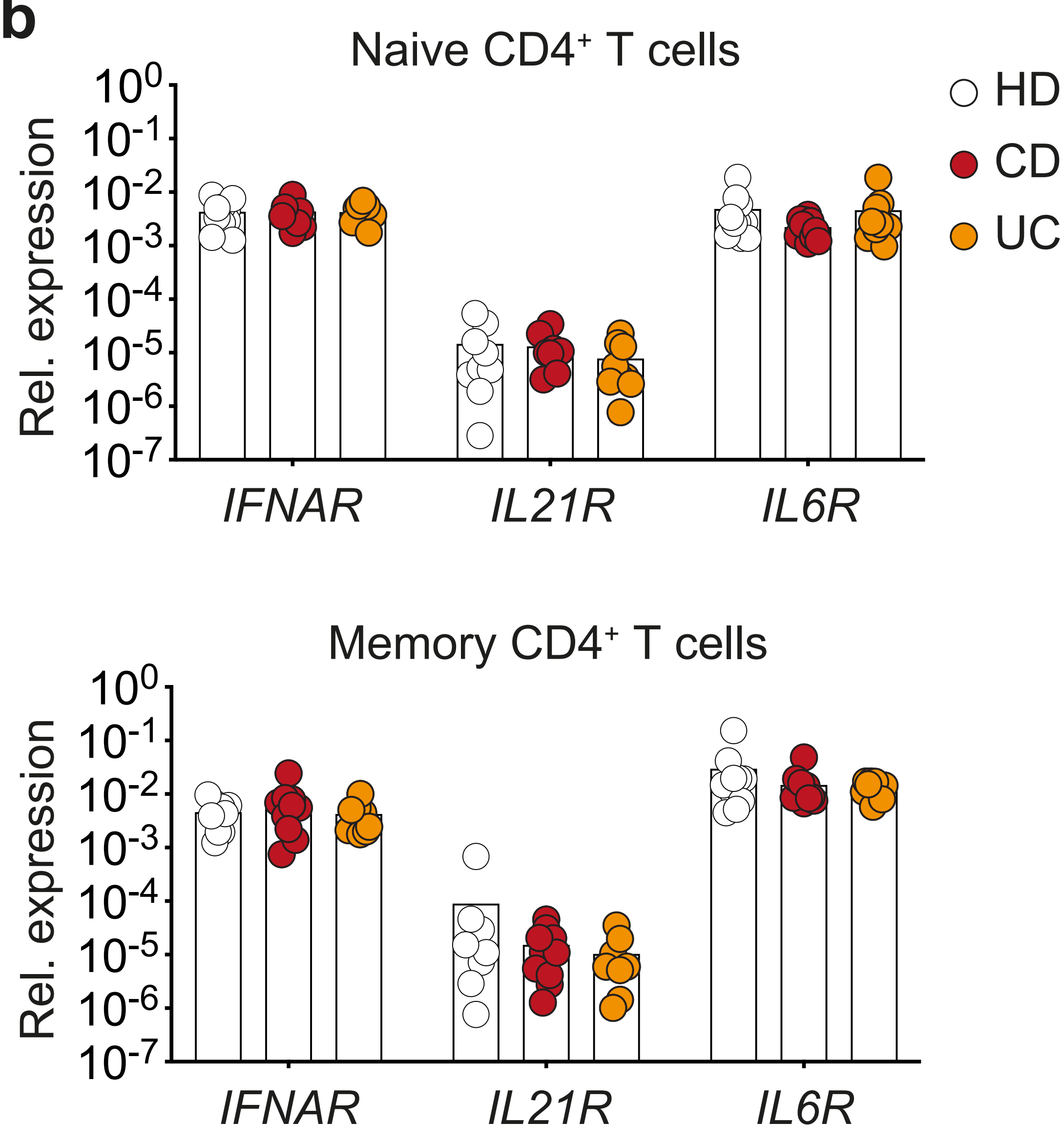

Supplement: Supplementary file 1 — Supplementary Materials [file 41385_2022_487_MOESM1_ESM.pdf]
